# Wood’s Physician’s Vade Mecum, and Visiting List

**Published:** 1877-11-20

**Authors:** 


Wood's Physicians' Vade Mecum, and Visiting List, arranged and prepared by H. C. Wood, M. D., Professor of Materia Medica, etc., in the University of Pennsylvania, etc

As a memorandum book and visiting list, this is among the best that has ever been prepared in this or any other country. J. B. Lippincott & Co., Philadelphia, Publishers.

